# O019. Headache as an emergency in children and adolescents

**DOI:** 10.1186/1129-2377-16-S1-A142

**Published:** 2015-09-28

**Authors:** Laura Papetti, Alessandro Capuano, Samuela Tarantino, Federico Vigevano, Massimiliano Valeriani

**Affiliations:** Headache Centre, Division of Neurology, Ospedale Pediatrico Bambino Gesú, Istituto di ricovero e cura a carattere scientifico, Rome, Italy

Pediatric and adolescence headache is one of the most common causes of access in Emergency Departments (ED). We reviewed the literature and we found that headache in ED is generally a benign condition that tends to be self-limited or resolves after an appropriate drug treatment. Causes of non-traumatic headache in ED include more frequently primary headaches (21.8-66.3%) and secondary benign headaches (35.4-63.2%), while secondary life-threatening headaches are less frequent (2-15.3%) (Table 1). The most frequent worrying conditions include ventricoloperitoneal shunt malfunction, central nervous system infections, brain tumors, hydrocephalus, pseudotumor cerebri and hemorrhage. In a pediatric ED, the primary objective is to recognize the serious life-threatening conditions requiring immediate medical care among the wide spectrum of headache diagnoses. The diagnostic approach starts with a thorough history followed by a complete physical and neurologic examination. The temporal features may be useful to classify headaches into four temporal patterns (acute, recurrent acute, chronic progressive, chronic non-progressive) that aid in reaching the etiological diagnosis. A normal neurological examination has been demonstrated to highly correlate with the absence of relevant intracranial processes in several pediatric studies. Neuroimaging should be considered in patients with recent-onset severe headache or change in the type of headache or with associated signs or symptoms suggestive for intracranial diseases. The therapeutic management of headache in ED depends on general clinical conditions of the patients and the presumable etiology of headache [1].

**Table 1:** Comparison of the studies about etiology of headache in ED * only patients with focal neurological signs at admission to ED.

	Kan et al [2]	Lewis et al [3]	Leon-Diaz et al [4]	Conicella et al [5]	Burton et al [6]	Scagni et al [7]	Massano et al* [8]
Number of patients	130	150	185	432	288	550	101
Age (years)	>18	>18	2-15	2-18	2-18	0-16	6-18
Secondary benign headaches (%)	63.2	59.6	60.5	35.4	63.2	38	--
Secondary life-threatening headaches (%)	15.3	14.9	4.3	4.1	2	4	9.9
Primary headaches (%)	10	18	24.3	24.5	21.8	56.7	66.3
Unclassified (%)	11.5	7	10.8	36	13	1.3	23.7
